# Chronic venous insufficiency and graduated compression stockings: analysis of public health system patients’ adherence to treatment

**DOI:** 10.1590/1677-5449.200034

**Published:** 2021-06-04

**Authors:** Francisco Eduardo Coral, Giovanna Golin Guarinello, Alice Pavanatto Cavassola, Ana Luiza Moraes Rocha, Marina Mosele Guidi, Hudson Pires

**Affiliations:** 1 Pontifícia Universidade Católica do Paraná – PUCPR, Curitiba, PR, Brasil.; 2 Hospital Santa Casa de Misericórdia, Curitiba, PR, Brasil.

**Keywords:** compression stockings, venous insufficiency, treatment adherence and compliance

## Abstract

**Background:**

Chronic venous insufficiency (CVI) is a pathology of great importance due to its high worldwide prevalence, affecting up to 80% of the population. Its incidence increases with age and is more frequent in females. One of the most important treatment options is compression therapy and the main method employed is wearing graduated compression stockings, which is considered the basic treatment for CVI regardless of the patient’s clinical classification. In clinical practice, treatment outcomes are impaired by patients not wearing the stockings properly.

**Objectives:**

To analyze the rate of adherence to wearing graduated compression stockings and to understand the problem of treatment non-adherence.

**Methods:**

Cross-sectional observational study conducted from June 2017 to January 2019, based on administration of questionnaires to patients at a SUS vascular surgery clinic at a teaching hospital, in Curitiba, PR, Brazil. Data were analyzed using the IBM SPSS Statistics v.20.0 computer program.

**Results:**

240 patients were analyzed. Mean age was 57.5 ± 12.9 (22 - 86) and 84.2% of the sample were female. 106 of the 240 patients analyzed (44.2%) were non-adherent with wearing compression stockings. Reasons for not wearing the stockings were: financial; pain; ignorance of the need to wear them; heat; and others.

**Conclusions:**

The adherence rate observed in the present study was 55.8% and the most prevalent reason for not wearing stockings was financial.

## INTRODUCTION

Chronic venous insufficiency (CVI) of the lower limbs is an extremely common condition,^1^ with prevalence that increases with age, and higher incidence among females.^2^^,^^3^ Its pathophysiology involves long-term intravenous hypertension^4^^,^^5^ caused by dysfunction of the venous system due to valve incompetence, with or without obstruction of venous flow.^2^^,^^5^^,^^6^ The final results are reflux of blood flow and venous stasis.^2^

Clinical status varies,^2^ ranging from esthetic changes to presence of signs and symptoms such as venous ulcers that provoke significant morbidity and sick days off work.^1^^,^^7^ Diagnosis of CVI is clinical, based on patient history and physical examination.^2^^,^^8^

The clinical classification of CVI comprises six classes. Class 0 encompasses cases in which there are no visible or palpable signs of venous disease; classes 1 and 2 are initial stages of the disease; and classes 3, 4, 5, and 6 constitute more advanced disease, with edema (3), skin changes (4) and healed venous ulcers (5) or active venous ulcers (6).^2^^,^^5^^,^^6^^,^^9^

One of the most important treatment options is compression therapy, which is considered the basic treatment for CVI irrespective of the patient’s clinical classification.^4^ Graduated elastic compression stockings (GECS) are a simple method of compression therapy with high efficacy.^9^ Their action is mechanical, provoking reduction in the diameter of veins, reducing reflux and venous pressure and increasing flow velocity and venous return.^3^ They thereby improve muscle pump function and reduce blood stasis.^1^^-^3 Benefits are limited to the period during which they are worn, since once removed the hemodynamic effects on the lower limb cease within approximately 1 hour.^10^

In clinical practice, the results of compression therapy are compromised by poor adherence to treatment, impacting the results.^1^^,^^9^ The objectives of this study were to assess the rate of adherence to wearing graduated elastic compression stockings and to understand the problem of treatment non-adherence.

## METHOD

This study was approved by the Research Ethics Committee at the Pontifícia Universidade Católica do Paraná (CAAE: 56980816.8.0000.0020; ruling number: 2.955.862). This is a cross-sectional observational study conducted from June 2017 to January 2019, enrolling 240 patients of both sexes, over the age of 18 years, with CVI in treatment at a Lymphedema and Angiodysplasia Clinic run by the Brazilian National Health Service (SUS - Sistema Único de Saúde) at a teaching hospital in the municipal district of Curitiba, Paraná, Brazil.

The exclusion criteria were presence of lower limb chronic obstructive arterial disease, decompensated heart failure, acute infectious or inflammatory processes involving the lower limbs, and follow-up drop outs. All study participants were aware of study objectives and methods and signed free and informed consent.

**Figure 1:**
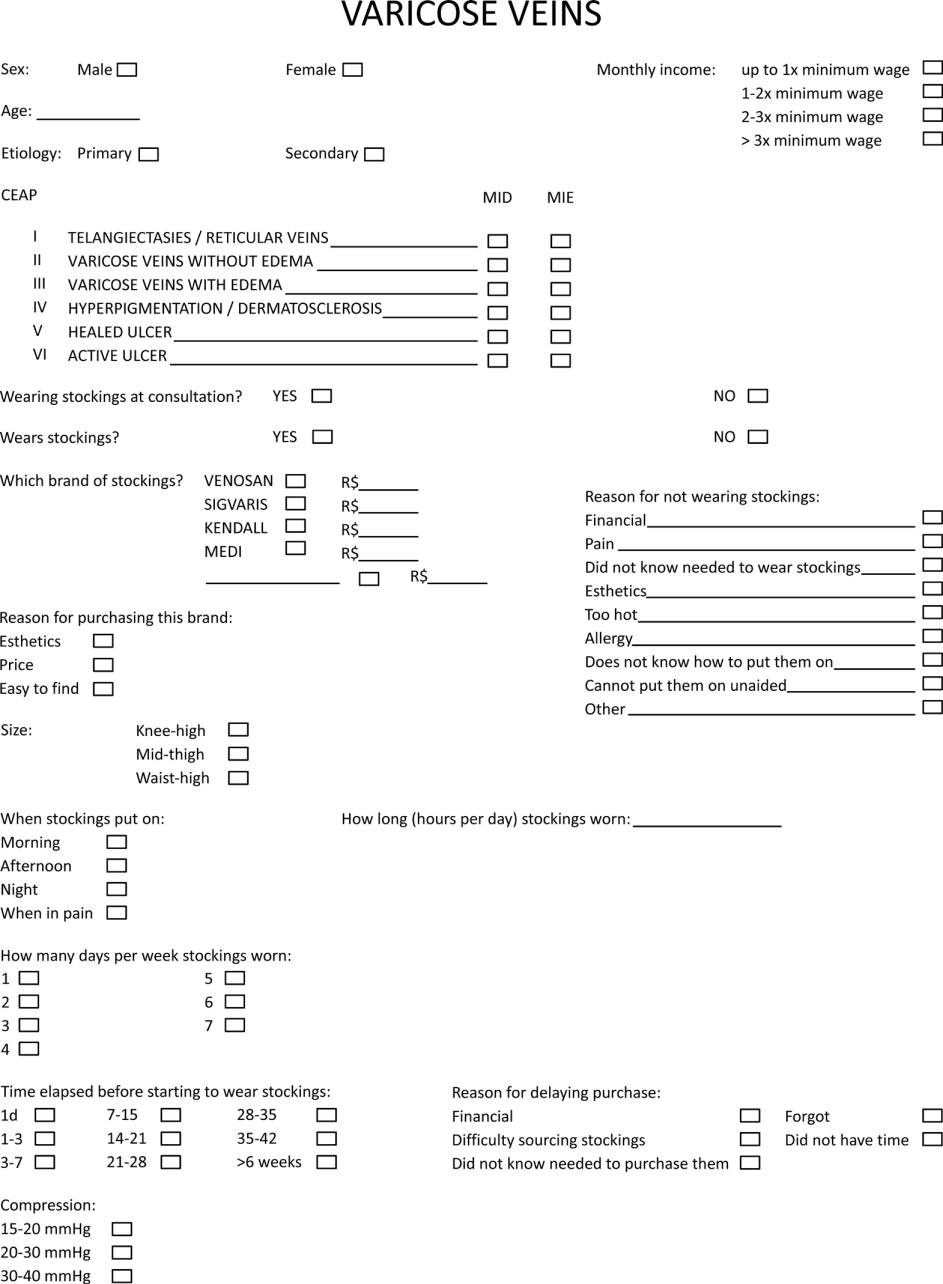
Questionnaire administered to study participants.

Data were collected during consultations to follow-up patients in treatment for CVI at the Lymphedema and Angiodysplasia Clinic. Information was collected using a questionnaire (Figure 1). Only the patient’s clinical classification was completed by the physician and all other questions were answered by the patient.

Adherence was defined as wearing the stockings for at least 7 hours per day on 5 days per week and patients were classified according to their most advanced clinical class. Data were stored in Microsoft Excel^®^ spreadsheets (Microsoft Corporation, Albuquerque, New Mexico, United States). The study results were expressed as means, standard deviations and minimum and maximum values (quantitative variables) or as frequencies and percentages (categorical variables). Associations between two categorical variables were assessed using the chi-square test. Results with p < 0.05 were considered statistically significant. Data were analyzed using IBM SPSS Statistics v.20.0 (IBM Corp, Armonk, New York, United States).

## RESULTS

A total of 240 patients were recruited, mean age was 57.5±12.9 (22-86), 202 (84.2%) patients were female and 38 (15.8%) were male. A total of 177 of the 240 patients analyzed (73.8%) reported wearing GECS and 63 (26.2%) did not wear them. The reasons that patients gave for not wearing GECS were financial issues (33.3%), pain (28.6%), ignorance of the need to wear them (19%), heat (6.35%), inability to put them on unaided or aided (6.34%), and others (6.35%) (edema; esthetics; didn’t want to wear them; and feeling that symptoms worsened).

Among patients who did wear stockings, the mean time wearing them per day in hours was 11.1±2.8 (2-18) and the mean number of days per week was 6.0±1.0 (1-7). Distributions of patients by age, sex, monthly income, clinical classification, whether they wear GECS at all, and adherence to treatment are shown in Table 1.

**Table 1:** General descriptive analysis.

**Variable**	**n (valid)**	**Classification**	**Result**
Age (years)	240		57.5±12.9 (22-86)
		20 to 40	31 (12.9)
		40.1 to 65	130 (54.2)
		> 65	79 (32.9)
Sex	240	Female	202 (84.2)
		Male	38 (15.8)
Monthly income	236	Up to 1 MMW	99 (41.9)
		1-2 MMW	97 (41.1)
		2-3 MMW	29 (12.3)
		> 3 MMW	11 (4.7)
Clinical classification	239	I	15 (6.3)
		II	57 (23.8)
		III	98 (41)
		IV	35 (14.6)
		V	20 (8.4)
		VI	14 (5.9)
Wears stockings	240	No	63 (26.2)
		Yes	177 (73.8)
Adherent	240	No	106 (44.2%)
		Yes	134 (55.8%)

Described by mean ± standard deviation (minimum–maximum) or as frequency (percentage). MMW = multiples of the minimum wage.

As mentioned above, for patients to be classified as adherent to treatment, they had to wear GECS for a continuous period of at least 7 hours per day, on 5 days per week, based on a standard working day. Therefore, 43 (17.9%) of the patients who stated that they did wear GECS patients were nevertheless considered non-adherent to treatment. As a result, 134 of the total sample of 240 patients were considered adherent to GECS treatment, equating to 55.8%, with a 95% confidence interval from 49.6% to 62.1%.

Analyses of individual variables considered valid data (without losses), since four patients did not inform their monthly income and one patient did not receive a clinical classification. With relation to factors associated with adherence, there were no statistically significant differences linked to age, sex, or clinical classification (p > 0.05), as shown in Table 2. The results of an analysis of the association between monthly income and adherence to wearing GECS are shown in Table 3, but the analysis did not detect statistical significance (p > 0.05).

**Table 2:** Analysis of variables × adherence.

**Variable**	**Adherence**		**Total**
	**Yes**	**No**	
**Sex**			
Female	115	87	202
	56.9%	43.1%	
Male	19	19	38
	50%	50%	
P = 0.478 (Fisher’s exact test, p < 0.05)			
**Age**			
20-40	19	12	31
	61.3%	38.7%	
41-65	71	59	130
	54.6%	45.4%	
> 65	44	35	79
	55.7%	44.3%	
P = 0.797 (chi-square test, p < 0.05)			
**Clinical classification**			
I	7	8	15
	46.6%	53.4%	
II to III	85	70	155
	54.8%	45.2%	
IV to VI	42	27	69
	60.9%	39.1%	
P = 0.528 (chi-square test, p < 0.05).			

**Table 3:** Monthly income × adherence.

**Income**	**Adherence**		**Total**
	**Yes**	**No**	
1 and 2 MMW	106	90	196
	54%	46%	
3 MMW	20	9	29
	68.9%	31.1%	
> 3 MMW	7	4	11
	63.6%	36.4%	

Value de p: 0.162 (Fischer’s exact test, p < 0.05). MMW= multiples of the minimum wage.

## DISCUSSION

There is a large body of medical literature suggesting that GECS treatment for CVI has considerable efficacy, such as, for example, a multicenter, prospective, double-blind, randomized clinical trial conducted in France by Benigni et al.,^4^ with 125 female patients, which demonstrated significant improvement in CVI symptomology at initial clinical stages associated with wearing low compression stockings (pressure at the ankle of 10 to 15 mmHg), compared with placebo stockings (pressure at the ankle of 3 to 6 mmHg). Vayssairat et al.^11^ conducted a similar study with 341 patients, also in France, in which the result was equivalent, with significant relief from symptoms associated with wearing compression stockings of the same grade.

However, despite the positive evidence in relation to wearing elastic stockings for CVI treatment, they are often worn too little or inappropriately. The rate of adherence observed in this study, at 55.8%, was higher than rates reported in the literature. A study conducted in the United States by Raju et al.^12^ found that 63% of patients who were prescribed elastic stockings did not wear them; the remaining 37% were classified as adherent, regardless of whether or not they wore them regularly.^12^ Ayala-García et al.^13^ analyzed a population in Mexico, observing that just 35.1% used compression therapy.

Another study, conducted in Poland by Ziaja et al.,^14^ found that 25.6% of the patients wore stockings and that adherence was greater among older patients and those with more advanced clinical classifications. These results corroborate what was seen in the present study, in which patients over the age of 65 years had greater adherence, although this observation was not statistically significant.

Another point that should be considered is that the sample in the present study comprised patients in treatment at a Lymphedema and Angiodysplasia Clinic, which is a factor that probably contributed to greater adherence. Ayala-García et al.^13^ analyzed 168 patients who had CVI from a non-specific vascular surgery clinic. Only 59 (35.1%) of the patients were adherent to treatment.^13^ Moreover, they observed a statistical difference between those who had and had not had some type of treatment for CVI, with those who had been treated being 3.3 times more likely to wear the stockings than those who had never had any previous medical care for vascular disease.^13^

The analysis of non-adherence to treatment and classification of participants as adherent or non-adherent was, however, compromised by the fact that there is no standard for how many days or for the number of hours they must be worn to define a patient as adherent. In one article, for example, Raju et al.^12^ explained that the concept of ‘compliance’ that they adopted was very broad, ranging from regular wearing, through wearing on some days, to infrequently wearing stockings.^12^

Analysis of the reasons for not wearing revealed a considerable divergence from populations studied in other countries. For example, the most common reason observed by Raju et al.^12^ were unable to specify a specific reason (30%), followed by not recommended by the physician, the impression that they were ineffective, too tight, hard to put on, and too hot. In the present study, the most common reasons were financial issues (33.34%), pain (18%), and ignorance of the need to wear them (12%).

With relation to the cost of elastic stockings, the study conducted in Poland by Ziaja et al.^14^ found that 33% of the patients stated that they did not wear them because of the high cost, which is a proportion that coincides with the rate found in this study (33.3%), whereas in the study conducted in the United States by Raju et al.,^12^ just 2% gave this reason. Although the costs are similar in all three countries, the present study was conducted with SUS patients (a free public-sector healthcare system), which may explain why this factor was the number one reason for not wearing stockings.

This study’s main limitation is the fact that it only enrolled SUS patients, and not a more homogeneous population sample. The importance of the data observed should be highlighted, as an aid to angiologists and vascular surgeons when prescribing GECS, providing a means of predicting and helping to solve difficulties with adherence to wearing them.

Moreover, it is worth stating that putting greater emphasis on explanations given during consultations would probably increase patient adherence. Some specialists in compression therapy point to low patient education as the root of the non-adherence problem, since many patients do not understand how to correctly employ compression therapy or its benefits.^15^^,^^16^ Involvement of physicians in patient education, explaining how the stockings should be worn and how to put them on, tends to increase adherence, increasing the benefits and improving the results of compression therapy.

## CONCLUSIONS

The adherence rate observed in the present study was 55.8%, which is higher than rates reported in the literature. We therefore infer and relate this finding to the following context: explaining the importance of wearing GECS to patients and following them up at a specialized clinic. The main reasons for not wearing stockings were financial issues, pain, and ignorance of the need to do so.
