# Supplementary material for: Chronic venous insufficiency and graduated compression stockings: analysis of public health system patients’ adherence to treatment
Source: J Vasc Bras. 2021 Jun 4;20:e20200034. doi: 10.1590/1677-5449.200034 (PMC8191186; doi:10.1590/1677-5449.200034)
Supplement: Translation

# Translation: Insuficiência venosa crônica e uso de meia elástica de compressão graduada: uma análise sobre a adesão ao tratamento em pacientes do SUS

**Authors:** Francisco Eduardo Coral, Giovanna Golin Guarinello, Alice Pavanatto Cavassola, Ana Luiza Moraes Rocha, Marina Mosele Guidi, Hudson Pires

**Affiliations:** 1 Pontifícia Universidade Católica do Paraná – PUCPR, Curitiba, PR, Brasil.; 2 Hospital Santa Casa de Misericórdia, Curitiba, PR, Brasil.

**Keywords:** meias de compressão, insuficiência venosa, cooperação e adesão ao tratamento

## Abstract

**Contexto:**

A insuficiência venosa crônica é uma doença de alta prevalência mundial, podendo chegar a até 80% da população. Sua incidência aumenta com a idade e é mais frequente no sexo feminino. Das opções terapêuticas, destaca-se a terapia compressiva, sendo a principal o uso de meia elástica de compressão graduada, considerado o tratamento básico para a insuficiência venosa crônica independentemente da classificação clínica do paciente. Na prática clínica, o resultado da terapia é prejudicado pela não adesão ao uso da meia.

**Objetivos:**

Avaliar a taxa de adesão ao uso da meia elástica de compressão graduada, assim como compreender a problemática da não aderência ao tratamento.

**Métodos:**

Estudo observacional transversal, realizado entre junho de 2017 até janeiro de 2019, mediante aplicação de questionário aos pacientes em ambulatório de cirurgia vascular do Sistema Único de Saúde (SUS), em um hospital-escola, em Curitiba, no estado do Paraná (PR). Os dados foram analisados com o programa computacional IBM SPSS Statistics v.20.0.

**Resultados:**

Foram analisados 240 pacientes. A média de idade foi de 57,5±12,9 (22-86); 84,2% eram do sexo feminino. Do total de pacientes analisados, 106 (44,2%) não aderiram ao uso das meias. As justificativas para o não uso foram: questão financeira, dor, desconhecimento da necessidade, calor e outras.

**Conclusões:**

A taxa de adesão encontrada no presente estudo foi de 55,8%, e o principal motivo para o não uso foi a questão financeira.

## INTRODUÇÃO

A insuficiência venosa crônica (IVC) dos membros inferiores é uma afecção bastante comum^1^, que tem sua prevalência aumentada com a idade e apresenta uma incidência maior no sexo feminino^2^^,^^3^. A sua fisiopatologia envolve a presença de hipertensão intravenosa de longa duração^4^^,^^5^ resultante de mau funcionamento do sistema venoso devido a uma incompetência valvular, com ou sem obstrução do fluxo venoso^2^^,^^5^^,^^6^. Os resultados finais são o refluxo sanguíneo e a estase venosa^2^.

O quadro clínico é variável^2^, desde alterações estéticas até a presença de sintomas e sinais que provocam morbidade significativa e perda de dias de trabalho, como a presença de úlceras venosas^1^^,^^7^. O diagnóstico da IVC é clínico, baseado nos dados da anamnese e do exame físico^2^^,^^8^.

A classificação clínica da IVC é composta por seis classes. Assim, 0 representa casos em que não há sinais visíveis ou palpáveis de doença venosa; 1 e 2, doença inicial; e os demais, 3, 4 e 5, característicos de doença mais avançada com edema (3), alterações de pele (4) e úlceras venosas cicatrizadas (5) ou ativas (6)^2^^,^^5^^,^^6^^,^^9^.

Das opções terapêuticas, destaca-se a terapia compressiva, considerada o tratamento básico para a IVC independentemente da classificação clínica do paciente^4^. Entre as terapias compressivas, está a meia elástica de compressão graduada (MECG), um método simples e de alta eficácia^9^. Ela atua mecanicamente provocando a diminuição do diâmetro das veias com redução do refluxo e da pressão venosa e o aumento da velocidade e do retorno venoso^3^. Assim, ela melhora a função da bomba muscular e reduz a estase sanguínea^1^^-^3. Os benefícios são limitados ao período do uso, uma vez que, após a sua retirada, o efeito hemodinâmico provocado sobre o membro inferior cessa em aproximadamente 1 hora^10^.

Na prática clínica, o resultado da terapia é prejudicado pela baixa adesão ao tratamento, prejudicando os resultados^ 1 ^^,^^ 9 ^. O objetivo deste trabalho foi avaliar a taxa de adesão ao uso da meia elástica de compressão graduada, assim como compreender a problemática da não aderência ao tratamento.

## MÉTODO

Estudo aprovado pelo Comitê de Ética em Pesquisa (CEP) da Pontifícia Universidade Católica do Paraná (PUCPR) – CAAE: 56980816.8.0000.0020. Parecer consubstanciado número: 2.955.862. Trata-se de estudo observacional transversal realizado entre junho de 2017 e janeiro de 2019, composto por 240 pacientes portadores de IVC com mais de 18 anos de idade, de ambos os sexos, atendidos no ambulatório de Cirurgia Vascular do Sistema Único de Saúde (SUS) de um hospital-escola no município de Curitiba, Paraná.

Os critérios de exclusão foram presença de doença arterial obstrutiva crônica de membros inferiores, insuficiência cardíaca descompensada, presença de processo inflamatório ou infecioso agudo nos membros inferiores e perda de seguimento. Todos os participantes do estudo estavam cientes da pesquisa e assinaram o termo de consentimento livre e esclarecido.

A coleta de dados ocorreu durante as consultas de retorno dos pacientes em acompanhamento por IVC no ambulatório de Cirurgia Vascular. As informações foram coletadas por meio de um questionário (Figura 1), sendo que o único campo preenchido pelo médico foi a classificação clínica, os demais tendo sido respondidos pelo paciente.

**Figura 1:** Questionário aplicado nos pacientes do estudo.

A adesão foi definida como o uso da meia por pelo menos 7 horas diárias em 5 dias na semana, e o paciente foi classificado de acordo com a classe clínica mais avançada. Os dados foram armazenados em planilha do Microsoft Excel^®^ (Microsoft Corporation, Albuquerque, Novo México, EUA). Os resultados obtidos no estudo foram descritos por médias, desvios padrões e valores mínimos e máximos (variáveis quantitativas) e por frequências e percentuais (variáveis categóricas). A associação entre duas variáveis categóricas foi avaliada considerando-se o teste de qui-quadrado. Valores de p < 0,05 indicaram significância estatística. Os dados foram analisados com o programa computacional IBM SPSS Statistics v.20.0 (IBM Corp, Armonk, Nova Iorque, EUA).

## RESULTADOS

Foram avaliados um total de 240 pacientes, a média de idade foi de 57,5±12,9 (22-86). Do total, 202 (84,2%) eram do sexo feminino, e 38 (15,8%), do sexo masculino. Dos pacientes analisados, 177 (73,8%) relataram fazer o uso da MECG, e 63 (26,2%) não faziam. As justificativas dos pacientes para o não uso da MECG foram questão financeira (33,3%), dor (28,6%), desconhecimento da necessidade (19%), calor (6,35%), não conseguir colocar com ou sem ajuda (6,34%) e outras (6,35%) (edema, estética, não querer usar e sensação de piora dos sintomas).

Nos pacientes que utilizavam, o tempo médio de uso diário em horas foi de 11,1±2,8 (2-18), e a média de dias na semana foi de 6,0±1,0 (1-7). A distribuição em idade, sexo, renda mensal, classificação clínica, uso da MECG e adesão é mostrada na Tabela 1.

**Tabela 1:** Análise descritiva geral.

**Variável**	**n válido**	**Classificação**	**Resultado**
Idade (anos)	240		57,5±12,9 (22-86)
		20 a 40	31 (12,9)
		40,1 a 65	130 (54,2)
		> 65	79 (32,9)
Sexo	240	Feminino	202 (84,2)
		Masculino	38 (15,8)
Renda mensal	236	Até 1 SM	99 (41,9)
		1-2 SM	97 (41,1)
		2-3 SM	29 (12,3)
		> 3 SM	11 (4,7)
Classificação clínica	239	I	15 (6,3)
		II	57 (23,8)
		III	98 (41)
		IV	35 (14,6)
		V	20 (8,4)
		VI	14 (5,9)
Faz uso da meia	240	Não	63 (26,2)
		Sim	177 (73,8)
Adesão	240	Não	106 (44,2%)
		Sim	134 (55,8%)

Descrito por média ± desvio padrão (mínimo-máximo) ou por frequência (percentual). SM = salário mínimo.

Como já citado, o paciente aderente deveria utilizar a MECG por um período contínuo de no mínimo 7 horas diárias por 5 dias na semana, considerando um período padrão de horário de trabalho. Dessa forma, apesar de relatarem fazer o uso da MECG, 43 (17,9%) pacientes também foram considerados como não aderentes ao tratamento. Sendo assim, do total de 240 pacientes, 134 foram considerados como aderentes ao uso de MECG, correspondendo a 55,8%, com intervalo de confiança de 95% dado por 49,6% a 62,1%.

Para a análise de cada variável, foram considerados os dados válidos (não perdidos), pois quatro pacientes não informaram a renda mensal e um paciente não foi classificado clinicamente. Em relação aos fatores associados à adesão, não houve diferença estatisticamente significante entre idade, sexo e classificação clínica (p > 0,05), conforme demonstrado na Tabela 2. A avaliação da associação entre a renda mensal e a adesão ao uso da MECG está demonstrada na Tabela 3, porém também não mostrou significância estatística (p > 0,05).

**Tabela 2:** Análise de variáveis × adesão.

**Variável**	**Adesão**		**Total**
	**Sim**	**Não**	
**Sexo**			
Feminino	115	87	202
	56,90%	43,10%	
Masculino	19	19	38
	50%	50%	
Valor de p: 0,478 (teste exato de Fisher, p < 0,05)			
**Idade**			
20-40	19	12	31
	61,30%	38,70%	
	
41-65	71	59	130
	54,60%	45,40%	
	
> 65	44	35	79
	55,70%	44,30%	
	
Valor de p: 0,797 (teste de qui-quadrado, p < 0,05)			
**Classificação clínica**			
I	7	8	15
	46,60%	53,40%	
II a III	85	70	155
	54,80%	45,20%	
IV a VI	42	27	69
	60,90%	39,10%	
Valor de p: 0,528 (teste de qui-quadrado, p < 0,05).			

**Tabela 3:** Renda mensal × adesão.

**Renda**	**Adesão**		**Total**
	**Sim**	**Não**	
1 e 2 SM	106	90	196
	54%	46%	
3 SM	20	9	29
	68,90%	31,10%	
> 3 SM	7	4	11
	63,60%	36,40%	

Valor de p: 0,162 (teste exato de Fischer, p < 0,05). SM = salário mínimo.

## DISCUSSÃO

Diversas literaturas médicas asseguram a grande eficácia ao tratamento da IVC com o uso da MECG, como, por exemplo, o ensaio clínico prospectivo multicêntrico randomizado e duplo-cego, realizado na França por Benigni et al.^4^, com 125 pacientes do sexo feminino, o qual demonstrou melhora significativa na sintomatologia da IVC em estágios clínicos iniciais com o uso de meias de baixa compressão (pressão no tornozelo de 10 a 15 mmHg) em comparação com meias placebo (pressão no tornozelo de 3 a 6 mmHg). Vayssairat et al.^11^ conduziram estudo semelhante, também na França, abrangendo 341 pacientes, no qual o resultado foi equivalente, com alívio significativo dos sintomas com a utilização de meias compressivas de mesma graduação.

No entanto, apesar das evidências positivas em relação ao uso de meias elásticas para o tratamento da IVC, seu uso é escasso ou inadequado. Os valores encontrados para adesão neste estudo, de 55,8%, foram superiores aos da literatura. No estudo de Raju et al.^12^, realizado nos Estados Unidos, 63% dos pacientes aos quais foi prescrito o uso da meia elástica não faziam o uso; os 37% restantes foram classificados como aderentes, independente de o uso ser regular ou não^12^. Ao analisar a população do México, Ayala-García et al.^13^ observaram que apenas 35,1% utilizavam a terapia compressiva.

Outro estudo realizado na Polônia, por Ziaja et al.^14^, encontrou uma percentagem de uso de 25,6% dos pacientes, sendo maior em pacientes mais velhos e que apresentavam uma classificação clínica mais avançada. Esse resultado corrobra o visto neste estudo, em que pacientes maiores de 65 anos eram mais aderentes, apesar de não haver significância estatística.

Outro ponto a ser levado em consideração é que a amostra do presente estudo foi composta por pacientes em acompanhamento no ambulatório de Cirurgia Vascular, sendo esse um fator que provavelmente contribui para a maior adesão. Ayala-García et al.^13^ analisaram 168 pacientes que apresentavam IVC, em um ambulatório não específico da Cirurgia Vascular. Do total, apenas 59 (35,1%) pacientes eram aderentes ao tratamento^13^. Além disso, foi observada uma diferença estatística naqueles que já tinham sido tratados de alguma forma para IVC, sendo esses 3,3 vezes mais prováveis de usar a meia, em comparação àqueles que nunca tiveram atenção médica prévia para doença vascular^13^.

A análise da não adesão ao tratamento e classificação dos participantes em aderentes e não aderentes, contudo, foi prejudicada pelo fato de não existir uma padronização quanto ao número de dias e de tempo em horas de uso para considerar o paciente aderente. Em seu artigo, Raju et al.^12^ explicaram que o conceito de *compliance*, por eles utilizado, é muito amplo, abrangendo uso regular, uso na maioria dos dias e uso infrequente^12^.

Ao levarmos em consideração os motivos de não uso, há uma grande divergência ao comparar com as populações de outros países. Por exemplo, no estudo de Raju et al.^12^, o principal motivo de não uso foi a incapacidade de determinar um motivo específico (30%), seguido de não recomendado pelo médico, sensação de inefetividade do tratamento, aperto, dificuldade de colocar e calor. No presente estudo, os motivos de não uso foram principalmente questão financeira (33,34%), dor (18%) e desconhecimento da necessidade (12%).

Com relação ao valor das meias elásticas, no estudo polonês realizado por Ziaja et al.^14^, 33% dos pacientes relataram que não fizeram o uso devido ao alto custo, valor que coincide com o encontrado neste trabalho (33,3%), enquanto, no estudo americano realizado por Raju et al.^12^, apenas 2% relataram essa justificativa. Apesar de apresentar valores similares nos três países, o presente estudo foi realizado apenas com pacientes do SUS, o que pode justificar esse fator como o principal motivo de não uso.

O presente estudo tem como limitações o fato de avaliar apenas pacientes do SUS, e não a população de uma forma mais homogênea. Ressalta-se a importância dos dados encontrados como auxílio aos angiologistas e cirurgiões vasculares no momento da prescrição das MECG como uma forma de prever e auxiliar nas dificuldades da adesão ao uso delas.

Ademais, vale enfatizar que o reforço na explicação feita em consultas provavelmente aumentaria a adesão dos pacientes. Alguns especialistas em terapia compressiva apontam a pobre educação do paciente como a raiz do problema de não adesão, pois diversos pacientes não entendem o correto uso e os benefícios da terapia compressiva^15^^,^^16^. O envolvimento do médico na educação do paciente, ao explicar o modo de uso e como vestir a meia, tende a aumentar a adesão e, portanto, a melhorar os benefícios e os resultados com a terapia compressiva.

## CONCLUSÃO

A taxa de adesão encontrada no presente estudo foi de 55,8% e superior às taxas encontradas na literatura. Assim, inferimos e relacionamos tal achado ao seguinte contexto: informação da importância do uso da MECG e acompanhamento no ambulatório especializado. Os principais motivos para o não uso foram a questão financeira, o relato de dor e a falta de conhecimento da indicação do uso.
